# Prevalence of Tracheostomy and Its Indications in Iran: A Systematic Review and Meta-Analysis

**Published:** 2019-04

**Authors:** Alireza Alidad, Alireza Aghaz, Ehsan Hemmati, Hussein Jadidi, Kayvan Aghazadeh

**Affiliations:** 1 Speech and Language Pathology, School of Rehabilitation Sciences, Tehran University of Medical Sciences, Tehran, Iran; 2 Research Committee and Department of Speech Therapy, School of Rehabilitation Sciences, Isfahan University of Medical Sciences, Isfahan, Iran; 3 Research Committee and Department of Biostatistics and Epidemiology, School of Health, Isfahan University of Medical Sciences, Isfahan, Iran; 4 Department of ENT, Tehran University of Medical Sciences, Tehran, Iran

**Keywords:** Tracheostomy, Indications, Prevalence, Systematic Review, Meta-Analysis

## Abstract

**Background:**

Tracheostomy is common among intensive care unit (ICU) patients. Reconsideration of tracheostomy indications in patients can be effective in modifying and reducing irrevocable patient complaints. The present study aimed to analyze the prevalence of tracheostomy indications and to estimate the prevalence of tracheostomy in Iran.

**Materials and Methods:**

In this systematic review and meta-analysis, scientific databases were searched from January 1990 to April 2018. The inclusion criteria were as follows: 1) use of the term “tracheotomy” in the title; and 2) studies conducted in Iran. On the other hand, the exclusion criteria were: 1) studies that did not specify the type of mechanical ventilation; 2) studies that did not quantitatively report the indications; 3) studies without access to the full-text; and 4) case studies, letters to the editor, and/or prefaces. Data were extracted from published reports. Our preliminary results included estimations of tracheostomy indications in Iran.

**Results:**

In the preliminary search, a total of 325 articles were found, 24 of which were considered eligible. Among 2860 patients who had undergone tracheostomy, 21 indications were identified. Decreased mental status, respiratory disease, and tumors were the most frequent indications. The prevalence of tracheostomy was 40.59% in Iran, with the highest and lowest rates reported in Birjand and Ardabil, respectively (136.50 and 6.63 in 100,000 people, respectively) based on the random effects model.

**Conclusion:**

The most prevalent indications in Iran are different from those reported in other countries. This difference may be due to the lack of trained medical personnel and available technologies.

## INTRODUCTION

Tracheostomy is the formation of a valve or opening into the trachea with the aim of facilitating air passage in upper airway obstructions and improving the discharge of pulmonary secretions or laryngeal storage in patients with long-term intubation (1, 2). The site of incision is usually between the second and third tracheal rings (3). The history of this surgery dates back to pre-Christianity in Greece (1, 4). According to previous studies, the prevalence of tracheostomy ranges from 6% to 65% (3). In this regard, Fischer et al. in a study from Switzerland showed that 60% of intensive care unit (ICU) patients had undergone tracheostomy in the second week (5), while in a study by Kluge et al. from Germany, the corresponding rate was 90% (6). On the other hand, studies from Iran have reported a prevalence rate of 24% (2).

In the past, the first indication for tracheostomy was the upper airway obstruction due to infectious diseases (7). However, considering the progress in vaccine development and use of antibiotics, infection is not the most prevalent indication for tracheostomy. In most previous studies, avoidance of prolonged intubation is the most common indication for tracheostomy (8, 9). However, the results of some studies are still controversial in this area. For instance, the most prevalent indications in some studies were respiratory diseases, head and neck tumors, and trauma to the jaws and skull, respectively (10). In some other studies, the most common indications were reported to be cardiac and pulmonary diseases, neurological disorders, and airway obstructions (7).

Since tracheostomy is an operation in which the upper airway is bypassed, it may pose serious risks to patients. Also, tracheostomy is one of the most common surgeries in the area of ear, nose, and throat (ENT); therefore, analysis of indications for tracheostomy can be effective in reducing the irreversible complications. The aim of the present study was to analyze the prevalence of tracheostomy indications and to estimate the prevalence of tracheostomy based on the data collected from Iranian studies.

## MATERIALS AND METHODS

### Search strategy and selection criteria

This systematic review and meta-analysis was conducted according to the PRISMA statement (11). From January 1990 to April 2018, databanks, including the Web of Knowledge, PubMed, Google Scholar, Science Direct, IranDoc, MagIran, and SID, were systematically searched, using the following keywords: “tracheostomy” or “tracheotomy”, “indication”, and “Iran”. The search included the online libraries of all medical universities in Iran. The keyword search in the websites of medical universities was conducted in both Persian and English languages. The Google search engine was also searched to obtain the full-text of the selected articles and to find more information about the subject under study. Moreover, to evaluate the results of keyword search, papers which were only suggested by the search engines were evaluated for their pertinence to the study.

Two authors conducted separate searches and appraised the title and abstract of each article based on the inclusion criteria. The inclusion criteria were as follows: 1) use of the keyword “tracheotomy” or “tracheostomy” in the title of the article; and 2) performing the study only in Iran. On the other hand, the exclusion criteria were as follows: 1) studies which did not properly define the type of mechanical ventilation (intubation or tracheotomy); 2) studies which did not report the indications in a quantitative manner; 3) studies with unavailable full-texts; and 4) case studies, letters to the editor, and prefaces. In the extracted studies, there was no limitation regarding the type of procedure (surgical or percutaneous) or time of surgery (late or early tracheotomy).

### Data extraction and risk of bias assessment

Using a standard form, the two authors (“AA” and “AAD”) separately extracted the data from the studies, including the authors’ names, location of studies, period of studies, number, gender, and average age of participants, and different indications for tracheotomy. Any disagreement between the authors was resolved through discussion with other researchers. The first author of the extracted studies was contacted in case of any ambiguities or to obtain additional information.

### Outcomes

The preliminary results of the present study were estimations of all indications for tracheostomy, based on studies on tracheostomy in Iran. Indications reported in each tracheostomy study were assessed. Since these indications varied in the extracted studies, the two authors agreed to identify similar causes, which referred to a specific disorder and to consider them collectively as one indication. The secondary results indicated the prevalence of tracheostomy. Since all studies were conducted during a specific period, the sample size for each study, relative to the population of the city where the study was conducted, could indicate the prevalence of this surgery in that particular city.

The prevalence of tracheostomy in each city was calculated by dividing the number of people undergoing tracheostomy by the city population in the year of its publication. To calculate the overall prevalence of tracheostomy, the total number of tracheostomies reported in the reviewed articles was divided by the total population of cities. The population of each city was determined based on the statistics published by the Statistical Center of Iran.

### Statistical analysis

Comprehensive meta-analysis (CMA) software was employed for data analysis. Variance of each study was calculated with regard to binomial distribution, and studies were combined considering the variance and sample size. To calculate the prevalence, 95% confidence interval was used to weigh each of the studies. A random effects model was also applied to combine studies with respect to their heterogeneity. Moreover, Cochran test and I2 index were utilized to evaluate the heterogeneity of studies.

### Funding source

There was no funding source in this study. Two of the authors had the required access to the necessary information. The decision to publish the article was made by all of the authors.

## RESULTS

### Selection of studies

**Figure 1.**
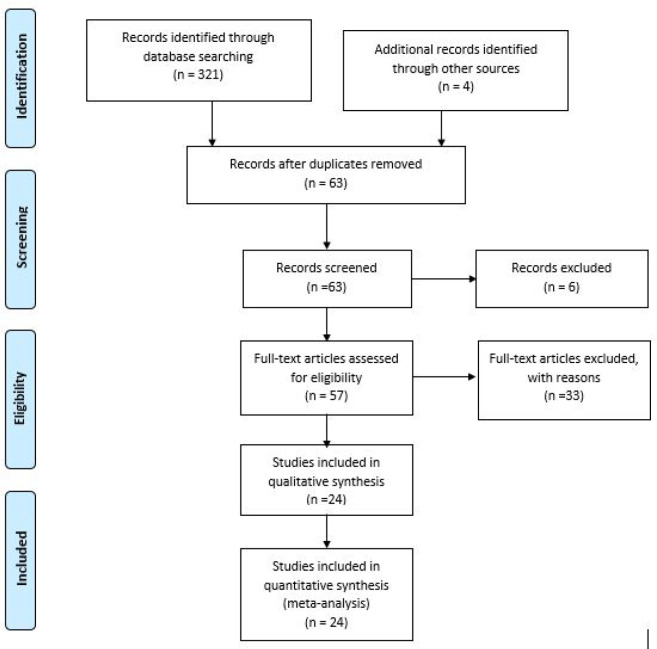
The Flowchart of selected articles

Based on the inclusion criteria, a total of 325 articles were found in the preliminary search of domestic and international websites (130 from Google Scholar, 29 from PubMed, 9 from ISI, 2 from Science Direct, 34 from IranDoc, 60 from MagIran, 57 from SID, and 4 from the digital libraries of medical universities in Iran). According to the exclusion criteria, 301 articles were eliminated, including 262 duplicate articles, 33 case reports, and six studies without the full-text manuscript. Finally, 24 full-text articles were included in the meta-analysis. The details of article selection are shown in Figure 1.

### Characteristics of studies

Twenty-four studies were published between January 1990 and April 2018. The selected studies were performed on 2860 patients with tracheostomy in 12 cities of Iran. In 17 articles, the average age of the patients was reported. Based on the findings, the average age of patients with tracheostomy was 49.2 years, and the age range of subjects in all 24 articles was 0–86 years. In 23 articles, 66.1% of the patients were male. These patients underwent tracheostomy due to 21 different indications. The participants’ age, gender, and publication date of each study are presented in Table 1.

**Table 1.** Trials of tracheostomy Indications meeting inclusion criteria

**Characteristics of studies**						**Indications or Causes of Tracheostomy (Number of Patients)**																				
	
**Study**	**Dates**	**Location (city)**	**Sample size (n)**	**Age(year)**	**Gender (male)**	**Respiratory disease**	**Inability to intubate**	**Neuromuscular disease**	**Depressed mental status**	**Pulmonary toilet**	**Head and neck surgery**	**Maxillofacil fractures or TMJ Ankylosis**	**Epiglottitis/supraglottitis**	**Tumor**	**Laryngeal problems**	**Foreign body**	**Blunt/penetrating neck trauma**	**Airway obstruction**	**Poisoning**	**Brain injury**	**Epilepsy and Tumor in brain**	**Diabetes**	**Hypertension**	**Septicemia**	**Suicide attempts**	**Heart problems**
	
Izadi (12)	1993 – 2004	Tehran	51	5/31	39	12		11						1	10	4	7	4								
Saleh (2)	2009 – 2011	Rasht	402	-	-				241											161						
Hasani (13)	2002– 2004	Tehran	126	-	84	11			50					29												
Farhanchi (14)	2009– 2010	Hamedan	36	48	25												25			7	4					
Vatandoust (15)	2013	Tehran	80	20 – 70	46				11		8			53						8						
Babaee (16)	2012 – 2014	Tehran	159	65/2	57	15														6		6				87
Karimpour (17)	2011 – 2015	Kermanshah	184	57/3	83	69		25												89						
Hashemian (18)	2009 – 2014	Tehran	360	51/7	233	237																		84		
Karvandian (19)	2009 – 2011	Tehran	100	55/5	68	65		35																		
Hashemzadeh (20)	2005 – 2010	Tabriz	50	33/48	32	2		31												11					6	
Shadab (21)	1993 – 1999	Tehran	131	0 – 86	125	16						9		17	81											
Hashemi (22)	1999– 2000	Shiraz	47	31/9	40	20					1		1		19	1	2									
Hemmati (23)	2013	Semnan	55	58/7	28				54																	
Hashemzadeh (24)	1991– 2000	Tehran	16	58/4	14										16											
Ghorbani (25)	2012 – 2014	Tehran	50	60/3	30															5		12	18			13
Javadi (26)	1996 – 2000	Tehran	140	0 – 85	103	14		1	56	3	1			44	4					17						
Bagheri (27)	2001 – 2010	Tehran	12	64/5	10	7								3	2											
Nikakhlagh (9)	2001– 2006	Ahvaz	151	47/8	104	7			53				5		74		11									
Malekzadeghan (28)	2010– 2016	Zabol	100	47/7	57	4	14		80						1											1
Amini (29)	1998– 2001	Ardabil	32	-	24								3		1				2	25	1					
Mofateh (30)	2000– 2007	Birjand	176	0 – 85	116	10			95	2	4			58												
Kiakojouri (21)	2001– 2006	Babol	96	47/4	66		9		71																	
Toutounchi (32)	1999– 2004	Tabriz	210	53/5	165				102						48											
Nemati (33)	2010– 2011	Rasht	96	50/8	77				56						21		1									

### Frequency of tracheostomy indications

**Figure 2.**
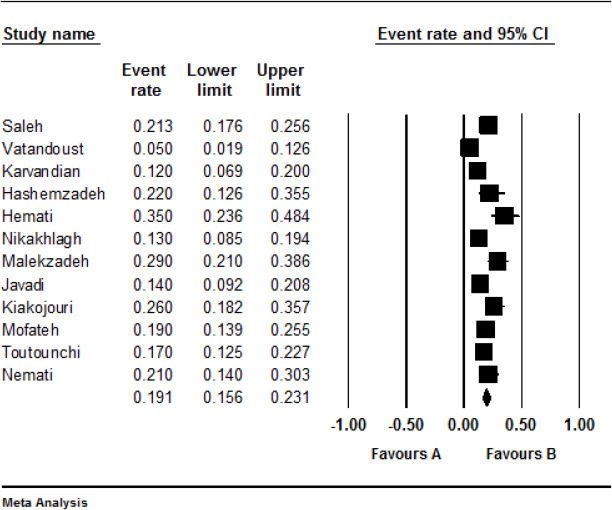
The percentage of tracheostomy cases due to the depressed mental status. The overall percentage of tracheostomy patients due to the depressed mental status in 12 studies was 0.19. The highest percentage was 0.35 in the Hemati study and the lowest rate was 0.05 in the Vatandoust study.

**Figure 3.**
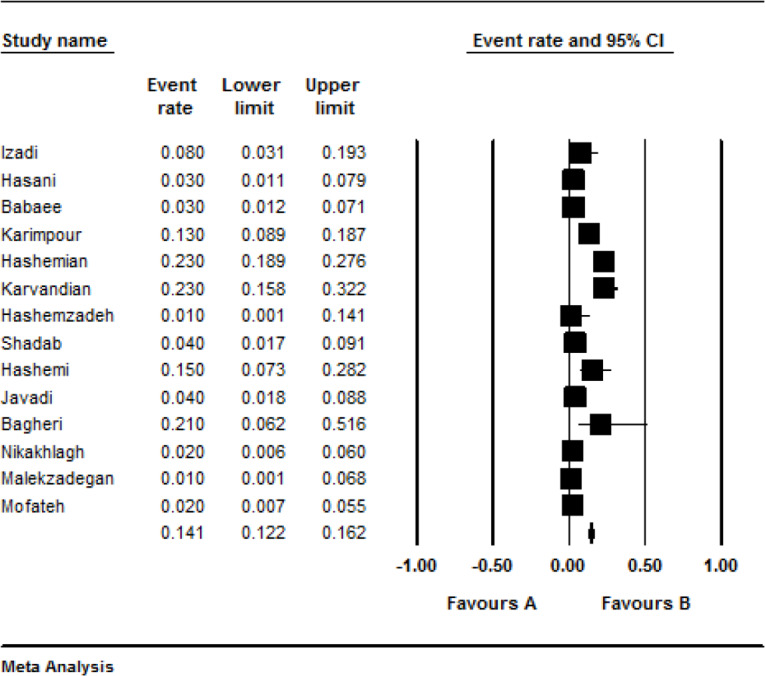
The percentage of tracheostomy cases due to the Respiratory disease. The overall percentage of tracheostomy patients due to the Respiratory disease in 14 studies was 0.14. The highest percentage was 0.23 in the Karvandian study and the lowest rate was 0.01 in the Hashemzadeh study.

**Figure 4.**
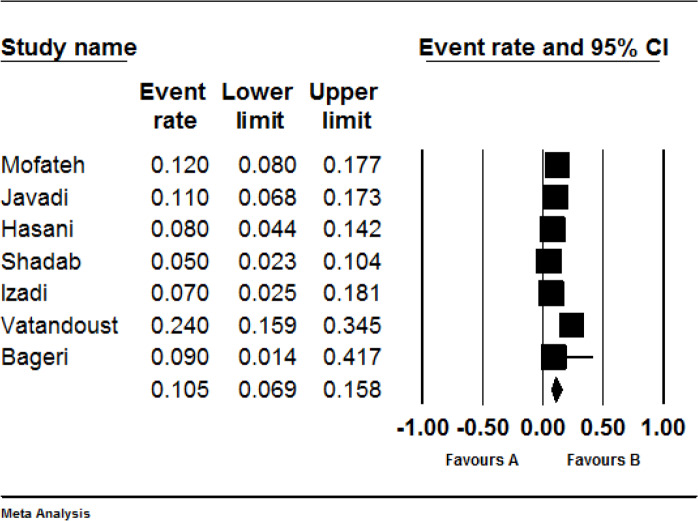
The percentage of tracheostomy cases due to the Tumor. The overall percentage of tracheostomy patients due to the Tumor in 7 studies was 0.105. The highest percentage was 0.24 in the Vatandoust study and the lowest rate was 0.05 in the Shadab study.

**Figure 5.**
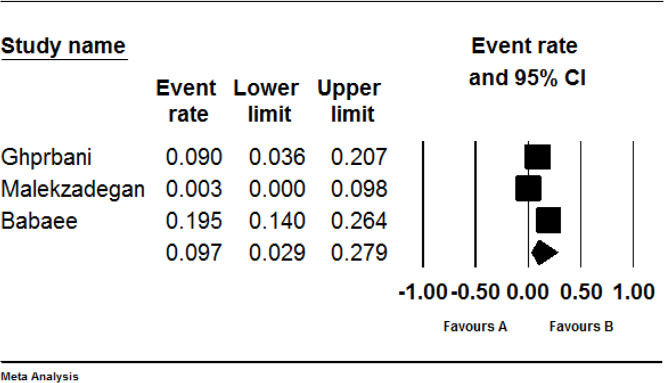
The percentage of tracheostomy cases due to the Heart problems. The overall percentage of tracheostomy patients due to the Heart problems in 3 studies was 0.097. The highest percentage was 0.195 in the Babaee study and the lowest rate was 0.003 in the Malekzadegan study.

**Figure 6.**
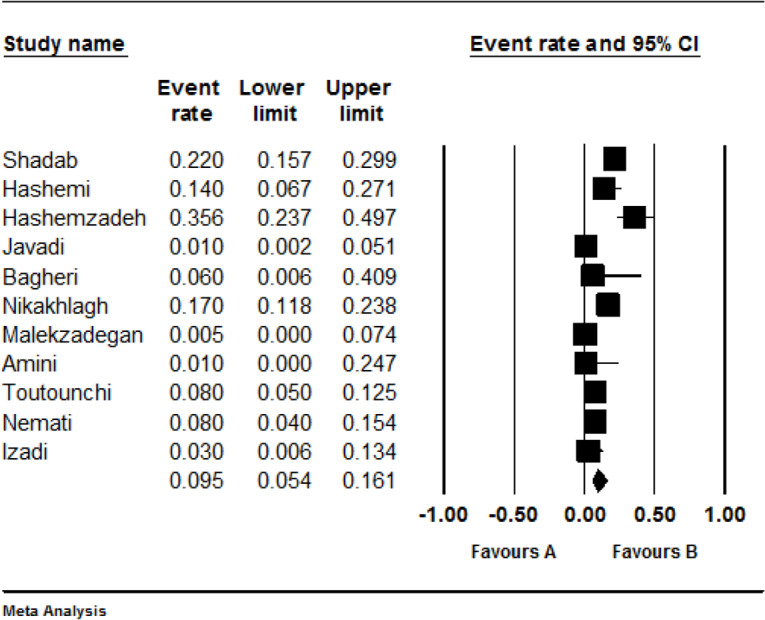
The percentage of tracheostomy cases due to the Laryngeal problems. The overall percentage of tracheostomy patients due to the Laryngeal problems in 11 studies was 0.095. The highest percentage was 0.356 in the Hashemzadeh study and the lowest rate was 0.005 in the Malekzadegan study .

In 24 selected studies, 21 indications were reported. The results of meta-analysis showed that the most common indications for tracheostomy were depressed mental status (19.1%), respiratory disease (14.1%), tumors (10.5%), cardiac problems (9.7%), and laryngeal problems (9.5%). These five indications comprised 62.9% of all indications (Figures 2–6). Depressed mental status (19.1%) was the most prevalent indication, and foreign body was the least common indication for tracheostomy. The frequency and percentage of these 21 indications are presented in Table 2. The obtained results were assessed using the random effects model at 95% confidence interval.

**Table 2.** Percentage and the frequency of different tracheostomy indications

**Indications**	**Frequency**	Percentage^*^
Depressed mental status	935	%19.1
Respiratory disease	489	%14.1
Tumor	214	%10.5
Heart problems	101	%9.7
Laryngeal problems	271	%9.5
Brain injury	329	%7
Neuromuscular disease	37	%5
Blunt/penetrating neck trauma	46	%4.5
Septicemia	84	%3.1
Inability to intubate	23	%2.7
Hypertension	18	%2.5
Diabetes	18	%2.3
Epiglottitis/Supraglottitis	9	%1.7
Head and neck surgery	14	%1.6
Epilepsy and Tumor in brain	11	%1.5
Pulmonary toilet	13	%1.4
Suicide attempts	6	%1.2
Airway obstruction	4	%0.65
Poisoning	2	%0.64
Extensive maxillofacial fractures	2	%0.55
Foreign body	1	%0.55

* This results are reported according to Meta-Analysis

### Rate of prevalence

**Figure 7.**
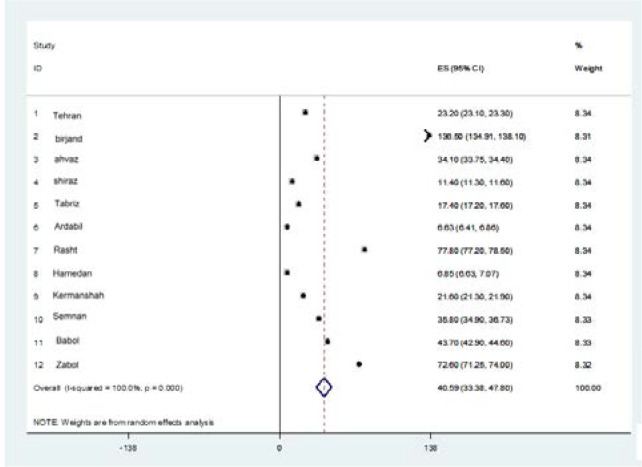
The rate of tracheostomy prevalence in every 100 thousand people and the 95% confidence interval in the considered studies based on the model of random effects. The middle point of each line shows the rate of prevalence, and the length of each line indicates the 95% confidence interval in each city. The rhombic sign shows the rate of prevalence combinations in the studies. The overall rate of tracheostomy prevalence in 24 studies was 40.59. The highest prevalence was 136.5 in Birjand and the lowest rate was 6.63 in Ardabil city.

Twenty-four studies reported the prevalence of tracheostomy in 12 cities of Iran. In each study, the rate of prevalence was calculated for each city as the number of tracheostomy cases in every 100,000 people relative to the population of cities. Regarding the considerable heterogeneity of studies (P<0.0001; I2=100%), the prevalence rate was measured for each study according to the random effects model. The prevalence of tracheostomy in the evaluated cities and the overall prevalence of tracheostomy in Iran are shown in Figure 7. The general prevalence of tracheostomy in Iran was 40.59 per 100,000 people (95% CI: 29.95–41.16), with the highest rate reported in Birjand (136.50 in every 100,000 people; 95% CI: 134.91– 138.10) and the lowest rate found in Ardabil (6.63 in every 100,000 people; 95% CI: 6.41–6.8) (Figure 7).

## DISCUSSION

The present study was conducted to estimate the indications and prevalence of tracheostomy in a systematic review of studies published in the last three decades in Iran. The results of eligible studies showed that the most common indications for tracheostomy in Iran were different from those reported in other countries. According to the present findings, the most common indications were depressed mental status (19.1%), respiratory diseases (14.1%), tumors (10.5%), cardiac problems (9.7%), laryngeal problems (9.5), and brain injury (7%). On the contrary, in a similar study, the most common indications were cardiopulmonary disease (32%) and neurological disorders (1%) (7).

In another retrospective review, prolonged intubation (35%), upper airway obstruction (28%), neurologic disorders, and craniofacial anomalies (12%) were the most prevalent indications for tracheostomy (34). Differences in the leading cause of tracheostomy are not exclusive to Iran, as every country has its own demographic pattern of health issues, different numbers of trained medical personnel, and different available technologies. In this regard, various reports have been recently published in England, suggesting that the most common indications were infections due to airway obstruction and need for long-term ventilation support (35).

Depressed mental status means loss of consciousness for any reason lead to intubation and artificial ventilator support, which results in tracheotomy if prolonged. Respiratory disease, as the second most common indication for tracheostomy in Iran, includes Subglottic Stenosis, Dysplasia, Pulmonary diseases, Asthma, Pneumonia, Croup, Angina, and Abscess. Tumors, such as Neoplasms of the larynx, thyroid, trachea, and esophagus, are the third most common etiologies in Iran. Brain injury includes Trauma, brain stroke, cerebral hemorrhage, cerebral hypoxia, and multiple traumas. Head and neck surgery includes restoration, cancer, and head and neck surgeries. Moreover, indications related to laryngeal problems include laryngeal cancer, laryngomalacia, laryngitis, and laryngeal papillomatosis.

The results of the present meta-analysis showed that the general prevalence of tracheostomy in Iran is 40.59 in every 100,000 people. According to this investigation, Birjand has the highest prevalence of tracheostomy among Iranian cities, while Ardabil has the lowest rate. Similar to other meta-analyses, the present study had some limitations. Systematic analyses were mainly performed among cross-sectional studies, and lack of cohort and case-control studies adversely affected the results. Therefore, it is essential to conduct further research in this area. In this study, classification of indications was based on the patients’ records before and after surgery. In addition to medical records, the tracheostomy surgeon can select the operation according to the patient’s condition. Moreover, two simultaneous diagnoses were suspected in many patients, which could affect the final decision for tracheostomy.

Finally, for estimating the prevalence rate of tracheostomy in Iran, we analyzed the results of studies conducted in 12 different cities according to the city population. In all of these cities, we had no access to the number of people with tracheostomy, and estimations were only based on the published articles. Therefore, regarding the lack of access to the number of people undergoing tracheostomy in these cities and non-consideration of other cities in Iran, the obtained results cannot be confidently generalized to represent all cities in the country, and caution must be taken while interpreting the results.

## CONCLUSION

According to studies conducted in the past 28 years in Iran, the most common indications for tracheostomy were decreased mental status, respiratory diseases, and tumors. These findings were not in line with the results of studies performed in other countries. Also, the present review showed that the general prevalence of tracheostomy is 40.59 per 100,000 people in Iran. However, to better understand the epidemiology of tracheostomy in Iran, more extensive studies with a larger sample size are necessary to provide more precise information.
